# Diabetic Ketoacidosis Precipitated by Henoch-Schonlein Purpura

**Published:** 2009-06

**Authors:** İlhan Asya Tanju, Ferhat Çekmez, Ozgur Pirgon, Ferhan Karademir

**Affiliations:** 1 *Department of Pediatrics, GATA Haydarpasa Medical Faculty, Istanbul, Turkey;*; 2 *Department of Pediatric Endocrinology, Konya Research Hospital, Konya, Turkey*

**Keywords:** Henoch-Schonlein purpura, type 1 diabetes, child, pancreatic involvement

## Abstract

Numerous investigations have been devoted to the search for environmental factors controlling the onset of autoimmune diseases. Pancreatic involvement, a rare complication of Henoch-Schonlein purpura, has been found mainly in adults, and it has not been reported in children. We present a case of a severe diabetic ketoacidosis in a child, a 7-year-old boy, following a typical clinical picture of Henoch-Schonlein purpura. Therapy with intravenous insulin resulted in resolution of the diabetic ketoacidosis and resolved the petechial rash of both legs.

## INTRODUCTION

Henoch-Schonlein purpura (HSP) is the most common vasculitis in childhood and is characterized by a systemic leukocytoclastic angiitis, mainly affecting the small vessels of the skin, joints, gastrointestinal tract, and more rarely, kidneys. Other organs, such as the brain, lungs, and scrotum, may occasionally be involved (1, 2). The etiology remains unknown, although many antigens, such as infecious agents, vaccinations, drugs, foods, and insect bites have been found to trigger HSP. The ability of superantigens to activate large numbers of T cells suggests that they may play a role in the course of autoimmune disorders. The principal pathogenic mechanism of type 1 diabetes (DM) is cytotoxic killing of beta cells of pancreas mediated predominantly by T cells. DM develops in genetically susceptible individuals and could be associated to several environment factors (3). The simultaneous association of HSP with DM has not been reported before in children, however, the association of HSP and DM has been reported several times in adults. Here we present a case of a severe diabetic ketoacidosis in a child following a typical clinical picture of HSP.

## CASE REPORT

**Figure 1:**
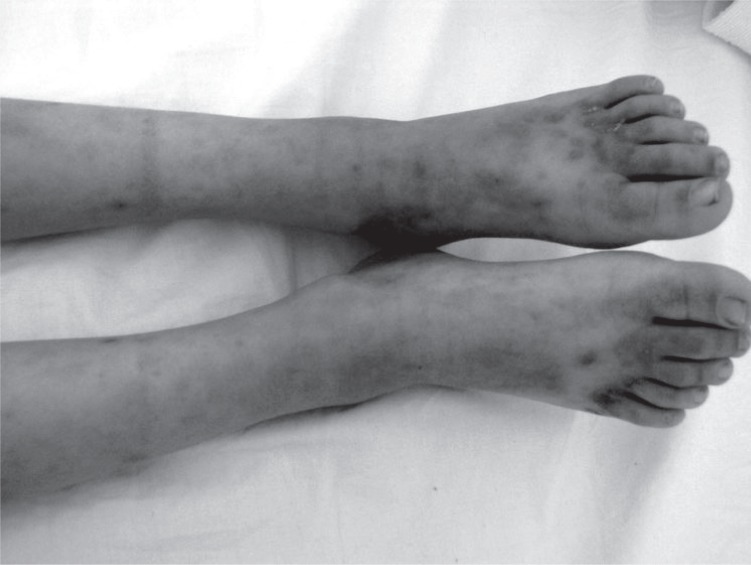
The child had petechial rashes on his abdomen and lower extremities that progressed to nonblanchable macules.

A previously healthy 7-year-old boy was brought to the emergency department by his mother with complaints such as vomiting, fatigue and colicky abdominal pain associated with diffuse, self-limiting arthralgias, and petechial rash of both legs. His medical history revealed having throat infection one month before admission. Complete blood count was within normal range for his age, as were his serum electrolytes. Blood examinations revealed an elevation of C-reactive protein and revealed both normal white blood cell and platelet counts. Serum IgA, C3 and C4 levels were all within normal limits and a urine examination also showed normal findings. Physical examination revealed mild proteinuria, hypertension and moderate anasarca (periorbital edema, ascites, and lower-extremity pitting edema). No medication and renal biopsy were performed because of the silent findings. However, two weeks later, after the admission for HSP, he complained of polydipsia and polyuria complaints and a physical examination revealed a 10-kg decrease in his baseline weight. There was no parental history of diabetes. In physical examinaTion weight: 16 kg (3rd percentile); height: 115 cm (25–50th percentile); pulse rate: 80/min; respiratory rate: 24/min; blood pressure: 100/60 mmHg; temperature: 36°C, his general state was poor and his consciousness was lethargic. Skin turgor and tonus were also diminished. There were petechial rashes on his abdomen and lower extremities that progressed to nonblanchable macules (Figure 1). He also had a fruity breath smell, dry mucous membranes, and tachypnea. Other examinations were normal. Laboratory studies yielded the following values (normal ranges were given in brackets): white blood cell count; 14.200/mm^3^ (6000–17500) with a normal differential formula; blood urea nitrogen, 9.34 mg/dL (5.7–20.1); serum creatinine, 0.6 mg/dL (0.1–0.9); albumin, 4.2 g/dL (3.5-5); sodium, 127 mmol/L (139–146); potassium, 4.51 mmol/ L (3.5–6.0); phosphorus, 2.8 mg/dL (4.9–7.9); and glucose, 27.83 mmol/L (3.3–5.0). Arterial blood gas values showed a pH of 7.10 (7.35–7.45); and bicarbonate: 8.4 mmol/L (21–28). Urine and serum ketones were positive. Urinalysis showed glycosuria. HbA1c was 11.7% (4.2-6.4). C-peptid level was 0.7 ng/mL (1.1-3.2). Insulin level was 4.28 μIU/mL (1.9-23). Autoantibodies including insulin, islet cell, glutamic acid decarboxylase were negative. Thyroid function tests were normal. Prothrombine time and partial thromboplastin time, immunoglobulin A, complement proteins C3 and C4, anti-DNA, antinuclear antibodies, and ASO levels were normal, as were serological tests for EBV and TORCH. Serum antigliadin and anti-endomysium antibodies were negative for the diagnosis of Celiac disease. Occult blood test in stool, urine and throat cultures were negative. The diagnosis of type 1 DM which presented with diabetic ketoacidosis accompanied by HSP was clinically made. A bolus of normal saline (20 mL/kg) was given intravenously, after which intravenous fluids were continued. Regular insulin via a continuous infusion at 0.1 IU/kg/h was started, and potassium was administered at 1 mEq/kg/day. The patient subsequently recovered and was followed by the Pediatric Diabetes Unit and given continued insulin treatment.

## DISCUSSION

The etiology of autoimmune diseases remains largely unknown but candidate etiologic factors include genetic abnormalities and infections. Although there are considerable data supporting the role of infections in a variety of autoimmune diseases, this role has been unequivocally established in only a few autoimmune diseases. The difficulty in establishing the infectious etiology of autoimmune diseases stems from several factors such as the heterogeneity of clinical manifestations in individual autoimmune diseases and the time interval between infection and autoimmune disease (4). Autoimmune or immunological disorders, such as HSP, SLE, Wegener’s granulomatosis, and Goodpasture disease frequently affect the organs. There are quite a number of reports in the literature of pancreatic involvement in various vasculitides, for example, PAN, Wegener’s granulomatosis, Takayasu arteritis and Kawasaki disease.

Bacterial infections have been implicated but other organisms including virus and mycoplasma have also been reported to precede HSP. As a result, at the capillaries, venules and arterioles of the target organs (such as skin, kidney and gastrointestinal system), neutrophil, eosinophil, and fibrin accumulate and so they cause leukocytoclastic vasculitis (5). In the vast majority of cases involving children and adolescents, type 1 DM is an autoimmune disease possibly triggered by a viral infection in a genetically susceptible individual (6). We speculated that HSP-triggered immunological abnormalities leading to insulitis may have led to the development of diabetes. In the presented patient, HSP developed subsequently to a throat infection, probably due to a viral agent and two weeks later, the patient was admitted clinically with the picture of diabetic ketoacidosis. Pathophysiologic mechanism of pancreatic involvement in HSP may be presumably a vasculitis of the small vessels especially within the pancreas leading to inflammation. As a pathogenetic mechanism, Samarkos *et al* proposed the production of superantigens by the infectious agent. Superantigens can activate a large number of T cells of different antigenic specificities with production of cytokines such as interleukin -1β, IL-2, IL-6, IL-8, and tumor necrosis factor-α (5,7). Cytokines may cause tissue damage or induce polyclonal production of immunoglobulin A and immunoglobulin G, resulting in immune complex formation (8). In our recent report, we reported that there may be an association between familial Mediterranean fever and type 1 DM explained by cytokine related damage (9). Insulitis has frequently been found in the early stage of IDDM, and is accompanied with infiltrations of small lymphocytes into pancreatic islets, which are mainly composed of T-lymphocytes, indicating that insulitis is attributed to abnormalities of cell mediated immunity, which can be triggered by viral infections such as Coxsacki B4. We suggest that cytokines may have a role in the pathogenesis of type 1 DM accompanied by HSP.

In adults, there are quite a number of reports in the literature of pancreatic involvement in HSP but not in children (10, 11). In terms of HSP and diabetes type 1, super-antigens may have a possible role in the pathogenesis. The other scenario might be that the child was manifesting early diabetes prior to the HSP development and that during the latter the diabetes became much more apparent. This, might, of course mean that the HSP had some type of triggering role, although not necessarily initiating the diabetic condition. In conclusion, there are several reports presenting “pancreatitis” and HSP in adults. In the present study we suggest that HSP also has a potential role in the subsequent development of diabetes in children.
